# The role of tumor volume in radiotherapy of patients with head and neck cancer

**DOI:** 10.1186/1748-717X-9-23

**Published:** 2014-01-14

**Authors:** Tomasz Rutkowski

**Affiliations:** 1 Department of Radiation Oncology, Maria Sklodowska-Curie Memorial Cancer Center and the Institute of Oncology, Gliwice Branch, Poland

**Keywords:** Tumor volume, Radiotherapy, Head and neck cancer

## Abstract

The assumption that the larger tumor contains a higher number of clonogenic cells what may deteriorate prognosis of patients treated with RT has been confirmed in many clinical studies. Significant prognostic influence of tumor volume (TV) on radiotherapy (RT) outcome has been found for tumors of different localizations including patients with head and neck cancer (HNC). Although TV usually is a stronger prognostic factor than T stage, commonly used TNM classification system dose not incorporate TV data. The aim of the paper is to refresh clinical data regarding the role of TV in RT of patients with HNC. At present somehow new meaning of TV could be employed in the aspect of modern RT techniques and combined treatment strategies. For larger TV more aggressive treatment options may be considered. In modern RT techniques escalated dose could be provided highly conformal or RT can be combined with systemic treatment increasing therapeutic ratio. In the study several reports estimating prognostic value of TV for patients with HNC treated with RT has been reviewed.

Due to substantially various reported groups of patients as to tumor site, stage of disease or treatment strategies, precise cut-off value could not be establish in general, but the significant association between TV and treatment outcome had been found in almost all studies. There is a strong suggestion that TV should supplement clinical decision in the choice of optimal treatment strategy for patients with HNC.

## Introduction

One of the principles of modern radiation biology is that a higher radiotherapy (RT) dose is needed to sterilize a higher number of clonogenic cells in larger tumors. The requirement for increased radiation dose for larger tumors is related also to other factors like hypoxia, clonal radioresistance or intercellular communication. While these factors are difficult to predict for given tumor, it has been reported that clonogen number increases linearly with the tumor volume (TV) [1,2]. In accordance to the theory, TV has been reported as an important factor influencing RT outcome of patients with various types of tumors including head and neck cancers (HNC). The commonly used TNM classification system for HNC does not reflect TV, incorporating linear dimension only for few tumor localizations. Compared to other known tumor response predictors, the TV appears to be specific and relatively easy to obtain. Modern radiation oncology requires the use of various imaging modalities to adequately identify and delineate the TV usually surrounded by normal anatomic structures. Along with this, improvement in segmentation algorithms and advances in computed technology occurred, giving the possibility for routine TV assessment. Due to precise estimation, TV may serve as a additional predictor for the choice between more and less aggressive treatment strategy.

At present somehow new meaning of TV could be employed. Modern RT techniques or combined treatment strategies (radiochemotherapy – CHRT) that could intensify therapy should be considered for patients with large TV. The aim of the paper is to refresh clinical data regarding the role of TV in RT of patients with HNC. Several studies on this matter has been reviewed and discussed.

## Material and methods

A computer literature search in MEDLINE was performed. For this purpose the following free-text terms were used: ‘Tumor volume’ or ‘Primary tumor volume’ or ‘Head and neck cancer/carcinoma’ or ‘Volumetric staging’, or ‘Volumetric analysis’. Additionally, extensive hand-searching of the references of all relevant studies was also performed.

### TV and TNM staging system

The stage of cancer at the time of diagnosis is crucial factor defining prognosis and remains the essential element in determining appropriate treatment based on the experience and outcomes of groups of prior patients with similar stage. In addition, according to AJCC, accurate staging is necessary to evaluate the results of treatments and clinical trials, to facilitate the exchange and comparison of information among treatment centers and to serve as a basis for clinical and translational cancer research [3]. The TNM system classification of malignant tumors was developed by Pierre Denoix between 1943 and 1952. It was originally constructed to assess only three basic indicators of anatomical spread: local tumor extent (T), locoregional nodal spread (N) and distant metastases (M). As outlined in *Purposes and Principles of Cancer Staging* 7^th^, although anatomic extent of disease remains central to defining cancer prognosis, an increasing number of nonanatomic factors about cancer and its host provide critical prognostic information and may predict the value of specific therapies. Revised AJCC staging algorithms gradually incorporate such factors. The TV seems to be one of nonanatomical factors representing properties of the tumor and showing significant impact on radiotherapy outcome but still not included to the classification system. Fundamental goal for radical (not palliative) radiotherapy is to kill the last surviving cancer cell, otherwise if one cell would survive local failure may likely be expected and therefore all of the dose would be wasted. Logically, total dose and its fractionation should be tailored to the initial number of tumor cells which strongly correlated with tumor volume (TV) rather than to the tumor diameter or stage T. If tumor diameter doubles (e.g. 1 to 2 cm or 2 to 4 cm) its volume increases by 8 fold and cell number increases by about one decade respectively. Design of dose fractionation for individual tumor depends on clinical, and recently more and more often, on molecular profile although TNM stage still plays the major role. However, there is relatively large variation of TVs within a given T stage N0M0 and it seems suboptimal to prescribe the same total dose to all tumors within a single T category.

Several authors have noted that TV is even better predictor of treatment outcome than TNM system or AJCC clinical stage [4-7]. Currently only about half of head and neck malignant tumors subsities are T-classified with the consideration of tumor dimension. In these cases the classification is based on a single dimensional measurement only. Such surrogate for TV seems to be not adequate. It was found that the difference in TV derived from diameter measurement and the computed-assisted perimeter method is large enough to have an impact on the response of the therapy [8]. Furthermore, staging based on single dimension measurement may be insufficient due to various clinical appearance of the tumor. The large tumor of epiglottis involving base of the tongue ((…) tumor invading mucosa of the region outside the supraglottic without fixation of the larynx) would be classified as T2. Small tumor of the glottis may infiltrate and destroy thyroid cartilage being classified as T4. Under the assumption that the number of tumor clonogens increases in direct proportion to TV [2] it seems to be clear that the second tumor, although T4, would be easier eradicated by radiation than previous one. With tumors staged clinically as T2 and T3 only 39% agreement between clinical and radiographic T stage was found by Kraas *et al*. [9]. The considerable variability of TV in a single T-stage category has been reported. Pameijer *at al*. found that among T3 tumors of different localization in head and neck region, TV variability was striking with variation exceeding 100%. Reported TVs for laryngeal, oropharyhngeal, hypopharyngeal tumors in T3 stage were from the range of 1.7-17 cm^3^, 10-41 cm^3^, 8.9-67.8 cm^3^ respectively. Moreover, the TV of T3 larynx and hypopharynx carcinoma showed a highly significant difference although the main TNM criterion for defining T3-stage in these subsities is the same, that is, vocal cord fixation. Much larger range of TV was reported for larynx and hypopharynx carcinoma defined by anatomical extent than for the oropharynx carcinoma which is defined by maximum diameter [10].

In the group of almost two hundred patients with T4 HNC of various sites with the mean TV of 64 cm^3^, Studer *et al*. used staging system based on three cut-offs to stratified TV and found it highly statistically significant for prognosis of RT outcome [11]. For T3 and T4 oropharyngeal tumors the TV ranges of 0-48 cm^3^ and 6.5-99.9 cm^3^ respectively were found [12]. The considerable range of TV inside the single T-stage is also true for early disease and generally smaller tumors. For 160 patients with T2 laryngeal tumors the range of TV from 0.15 cm^3^ to 21.68 cm^3^ has been reported [13]. For T1 and T2 hypopharyngeal tumors TV ranges were respectively as follows: 0.5-3.02 cm^3^ and 1.13-9.38 cm^3^[14]. The TV range of 0-32.5 cm^3^ for T2 oropharyngeal tumors was observed [12]. For 125 patients with T2 nasopharyngeal carcinoma the TVs were from 1.4 cm^3^ to 60.4 cm^3^[15].

As it can be seen, the limitation of TNM classification system is that it categorizes tumors with different volumes into the same stage. Use the pure anatomic extent of disease in defining treatment for patients with HNC seems to be not sufficient in many cases and clinicians should supplement therapeutic decision about optimal treatment with TV data.

### TV measurement

Two-dimensional approach to radiotherapy, CT beyond the rich in daily clinical practice or variation in TV measurement techniques have prevented TV assessment procedure from being routinely used in a clinical setting in the past. Modern radiation oncology is based on intensity-modulated radiation beam that may precisely sculpt the radiation dose to target volumes of almost any shape. In this framework computed tomography (CT) has become the reference imaging modality for treatment planning. It has intrinsic information on the electronic density of the various tissues, which is used for dose calculation algorithms. It is also widely available and does not cause geometric distortion. Due to that, pretreatment contrast-enhanced CT scans are the most common support for TV assessment. Some limitation of CT however should be mentioned: uncertainty in precise tumor delineation on CT scans usually associated with motion artifacts, crudeness of volume estimation given large slice thickness, presence of dental fillings or inherent difficulty in differentiating tumor from oedema or biopsy changes. In retrospective studies up to 40% exclusion rate of CT scans due to uncertainties in CT quality has been reported [16]. The degree of motion artifacts may be influenced by patient-controlled factors such as swallowing, coughing, breathing manner or movement. Due to technological advancement of multidetector spiral scanners less motion artifacts and potentially thinner slices could be obtained. Technical aspects of obtaining CT scans may differ for various tumor localization. For early stages of laryngeal tumors it is important to align properly the CT cuts with anatomic landmarks to be in plane with the true vocal cords.

The volumetric analysis depends on a hand-drawn region of interest. Whether this process is performed by radiologist, radiooncologist or technician, the element of subjectivity could not be avoid. This is an issue for potential interobserver and intraobserver error. To minimize this inaccuracy, the measurement should be done by single, trained observer [17]. The inter- and/or intra-operator variability is tried to be overcome also by semi-automated or automated systems. Errors encountered by computered based techniques exclude the problem of operator experience and are classified as systemic errors.

The definition of tumor boundaries on CT may carry on some difficulty in precise because separation the oedema and tumor from each other is complex. Usually the area of peritumor oedema is excluded from the region of interest (TV), but also no attempt to differentiate these two areas has been reported [18]. MR imagining, although not effective in differentiating edema form tumors, is more sensitive than CT in distinguishing tumors (together with peritumoral edema) from normal adjacent structures. MR also has been shown to be more accurate than CT in evaluating soft-tissue or bone extension [19]. It seems to be of special concern for some tumor sites (nasopharynx) where exclusion of invasion into the skull base may be essential. For oropharyhngeal and hypopharyngeal cancers primary TVs showed by CT and MR imaging did not exhibit significant differences [20]. MR does not carry any intrinsic information on electronic density mapping what precludes its use as the sole imaging modality for treatment planning.

Unlike CT and MR, fluorine 18 (^18^ F) fluorodeoxyglucose (FDG) positron emission tomography (PET) provides not only anatomy imaging but also non-invasive functional one, reflecting functional and biological metabolism. For treatment planning purposes, it was shown that the combined use of CT and FDG PET in lung tumors resulted in an improved delineation of the TV substantially reducing the irradiated lung volume [21]. PET does not provide any accurate information on the contours of the infiltration what precludes its use as a sole imaging modality for HNC treatment planning. Together with CT incorporates both, anatomic localization and functional information.

The results from recent studies of HNC indicate that high FDG uptake in the primary tumor, typically characterized as a standardized uptake value (SUV) may predict treatment outcome [22]. It is not clear however at that time, if higher SUV, greater than median one, or metabolic TV, tend to be better predictor [22,23]. It appeared that if TV is contoured in the pharyngolaryngeal area on CT scans, MRI scans and PET images, no significant difference between TV obtained at two first techniques is observed. The TV assessed by PET is smaller with the difference from 28% to 37% comparing to CT and MRI. Non of these TVs is totally overlapped with each other. Only up to 20% of the TV assessed from anatomic imagine (CT, MRI) could be observed with PET. Of interest, in comparison with surgical specimen of previously scanned tumors used as a reference, all the imaging modalities overestimated the original TV [20].

### TV and treatment results

#### Prognostic cut-off values for TV stratification

In most series cut-off levels of TV were based on objective criteria as a mean or quartiles (median) [13,24-26] or on subjectively estimated values of TV, also called “optimal cut-off” [9,14,18,27-30] or ranges of these values according to frequency of failure [6,11,31-33]. The site of the tumor determines TV as well. For laryngeal tumors cut-off values from the range of 4-6 cm^3^ were the most often used [9,18,30]. Hermans *et al*. stratified TV of glottic cancer for 5 classess (<1 cm^3^, 1-2 cm^3^, 2-4 cm^3^, 4-8 cm^3^, 8-16 cm^3^) [31] and supralaryngeal cancer for classes each 2 cm^3^ (range: <2 cm^3^ - >16 cm^3^) [32]. For relatively large series of 160 patients with T2 laryngeal cancer, median TV for those with supraglottic tumors was significantly higher than for glottic onces (5.5 cm^3^ v 0.8 cm^3^, p < 0.0001). For analysis, TV in that series was stratified according to quartiles [13]. Pamijer *et al*. stratified TV of patients with early hypopharyngeal tumors by optimal cut-off value of 6.5 cm^3^[14]. For nasopharyngeal cut-off value of 15 cm^3^ was often used [15,34]. Hermans *et al*. stratified tonsillar tumors in volume classes according to the quartiles: <6 cm^3^, 6-14.5 cm^3^, 14.5 – 31.2 cm^3^, >31.2 cm^3^) [25].

Reported thresholds of TV were also depended on treatment modality – usually larger for CHRT (>20 cm^3^) than for RT alone. The median value of 32.79 cm^3^ was used to stratified risk in patients with advanced oropharyhngeal cancer treated with CHRT [27]. Studer *et al*. for similar group of patients used four volume cut-offs: 1-15 cm^3^, 16-70 cm^3^, 71-130 cm^3^, >130 cm^3^[11]. Others used TV from the range of 22.8-112 cm^3^ for stratifying the risk of patients with advanced tumors of various localization treated with CHRT [6,7,24,26,28,29].

#### TV and RT treatment outcome

Most of the volumetric studies in patients with HNC have been reported for small groups of patients mixed as to tumor site, stage or treatment methods. Kraas *et al*. reported 28 patients with supraglottic cancer in various T- stage treated with RT. Median TV was 3.1 cm^3^ in this group. Local control (LC) rate at 2 years were 67% and 43% for TV cut-off at 6 cm^3^ (p = 0.07) and 20% and 70% for TV cut-off at 8 cm^3^ (p = 0.007). Mean tumor volumes for patients with or without recurrences were 10 cm^3^ and 3.4 cm^3^ respectively. Pre-epigoltic space invasion, subsite of disease or vocal cord mobility were not found to have an impact on local control rate [6]. Mancusco *et al*. in a study of 63 patients with T2-T4 supraglottic cancer found LC rates of 89% and 52% (p = 0.001) when tumors were less than 6 cm^3^ or ≥6 cm^3^, respectively. Moreover, the likelihood of maintaining laryngeal function also varied with TV: 89% for tumor less than 6 cm^3^ and 40% for tumors ≥6 cm^3^ (p < 0.001) [18]. Hermans *et al*. reported a significant correlation between TV classes and local control within the glottic T1 category (p = 0.006) in the group of 61 patients with T1-T4 glottic tumors [31]. In a group of 47 patients with T2-T3 glottic or supraglottic cancer reported by Hamilton et al. the TV ranged from 0.2 cm^3^ to 16.64 cm^3^ with the mean of 3.5 cm^3^. For all the group, those with TV of less than 3 cm^3^ recurred in only 22% of cases whereas there was a 65% recurrence rate in tumors greater than 3 cm^3^ (p = 0.003). For glottic cancer such cut-off value was at TV of 1 cm^3^. T-stage, preepiglottic space invasion, tumor location (glottic vs supraglottic) or vocal cord mobility were not found to correlate with local recurrence rate [35]. The TV implications was reviewed also in the group of 55 patients with T2,T3 either glottic or supraglottic cancer treated with RT or surgery. The TV >4 cm^3^ was a predictor of local failure in the subgroup of T2 patients treated with RT. This volume effect was not abolished with increasing radiation dose. No TV was identified to be a predictor of locoregional control in the group of patients surgically treated. T and N stage were not independent predictors of tumor control [30]. Mukherij *et al*. reported results of 28 patients with T2 glottic cancer. The study is one of few where the relationship between TV and RT outcome for laryngeal tumors was not found [36].

Contrary to this, the strong correlation between TV and RT outcome was found in the group of 115 patients with T2 glottic cancer treated exclusively with RT between 2002 and 2009. There was considerable, 10 folds difference between the smallest and the largest TV within a single, T2 classified tumors (median: 2.2 cm^3^, range: 0.16 – 17 cm^3^). Six time increase in the TV resulted in more than 3 time decrease in the LC. For TV ≤0.7 cm^3^ 3-year LC was 83%, 70% for TV of 0.7-3.6 cm^3^ and 44% for TV of 3.6-17 cm^3^ respectively. Independently of other factors, tumors with TV larger than 1.6 cm^3^ had significant, 3 times higher risk of local failure than smaller ones (HR 3.21, 95% CI 1.07 – 9.63, p = 0.03). Analysis of the total dose vs. initial TV has shown that within the T2 glottic cancer larger tumors with TV of about 5 cm^3^ (2-2.5 cm in diameter with 10^10^ cancer cells) need an extra 6.5 Gy to achieve similar 3-year LC as small tumors with 0.5 cm^3^ (~1 cm in diameter with 10^9^ cancer cells) [37]. Hermans *et al*. reported on 103 patients treated with definitive RT for supraglottic cancer. TV significantly influenced local control in univaraite analysis but in multivariate one, only pre-epiglottic space invasion and subglottic extension were found to by significant. TV was however the strongest independent predictor of locoregional failure (p < 0.01) [32].

Pameijer *et al*. reported on 23 patients with T1 and T2 carcinoma of the pyriform sinus treated with definitive RT between 1984 and 1993. Local control was observed in 89% of patients with TV ≤6.5 cm^3^ compared with 25% for patients with TV >6.5 cm^3^ (p = 0.02) [14].

There were only few studies considering TV in patients with oropharyngeal cancer treated exclusively with RT showing conflicting results. Studer et al divided large group of 277 patients with oropharyngeal cancer who underwent definitive IMRT into four subgroups according to TV (1-15 cm^3^, 16-70 cm^3^, 71-130 cm^3^, >130 cm^3^). Such volumetric staging appeared to be the most reliable in predicting treatment outcome in that group of T4 patients [38]. Hermans et al reported on 112 patients with tonsilar squamous cell carcinoma treated with definitive RT before 2001. TV was found to predict local control significantly when stratified by quartiles but not within T2-T4 stages. Multivariate analysis revealed that T stage significantly impacted local control whereas TV did not [25]. In another series result of analysis of 114 patients with T2-T4 oropharyngeal cancer treated between 1983 and 1995 with definitive RT or additionally with induction chemotherapy was reported. Multivariate analysis revealed that TV did not influence local control but T-stage significantly impacted this end point [12].

Also for nasopharyngeal carcinoma TV appeared to be significantly related to RT results. In the group of 308 patients with the median TV of 22 cm^3^, 3-year LC was 97% and 82% for patients with TV <15 cm^3^ and ≥15 cm^3^ respectively (p < 0.01). The TV remained an independent prognostic factor for the LC in multivariate analysis with the increase by 1% for every 1 cm^3^ increase in TV [15]. Sixty seven patients with T1 and 49 patients with T2 nasopharyngeal carcinomas treated with definitive RT between 1989 and 1991 were reported with the respect to TV. The median TV was 7.6 cm^3^ and 22.7 cm^3^ for these groups respectively. The 5-year local control rates were 93% and 82% for those with tumor ≤15 cm^3^ and >15 cm^3^ respectively [34].

#### TV and CHRT treatment outcome

Definitive CHRT has become a common option for the treatment and accepted as a method of organ preservation for patients with locally advanced HNC. This combined modality treatment has been associated with greater rates of toxicity. Careful patient selections seems to be crucial to optimize the therapeutic ratio for such modality. The TV measurement may provide valuable information to guide treatment decision. The potential goal of using TV as an outcome predictor is to identify patients who are unlikely to benefit from CHRT. These patients can be spared the intensive, toxic CHRT treatment regimes or can be considered for alternative treatment options (induction CHT, altered fractionation RT or combination of RT with other drugs like monoclonal antibodies or hypoxic cell radiosensitizers). The TV assessment may also help stratifying patients for clinical trials. For the group of patients for whom a favorable response is predicted, strategies can be developed to decrease toxicity and side effects. The prognostic value of TV for such patients has been reported by some authors and shows that the TV is a better predictor of outcome than either T or N stage. In a group of 78 patients with locally advanced carcinoma of larynx or pharynx the TV of 35 cm^3^ was found as an optimal cut-point. Seventy one percent of patients with a TV <35 cm^3^ were tumor-free at 5 years compared with only 41% of patients with TV >35 cm^3^ (p = 0.01). The 5-year overall survival (OS) rate for patients with TV <35 cm^3^ was 84% vs. 42% for the patients with TV >35 cm^3^ (p < 0.001). Similarly, the 5-year progression-free survival for patients with TV <35 cm^3^ was 61%, but it was only 33% for those with TV >35 cm^3^ (p = 0.01). On multivariate analysis TV remained statistically significant predictor of progression-free survival and OS while T and N stage did not. Neither the nodal disease volume nor the total sum of all disease (primary and nodal) was a statistically significant prognostic factor [7]. Chen *et al*. found that for patients with advanced stages of hypopharyngeal tumors without bulky lymph nodes treated with definitive CHRT, TV less than 30 cm^3^ form a favorable group for which definitive CHRT with laryngeal preservation scheme may be suitable [29]. In another study patients with oropharyhngeal tumor (mean TV: 24 cm^3^) had significantly better 3-year locoregional control comparing to patients with oral cavity tumor (mean TV: 41 cm^3^) (p = 0.01). For all the group the threshold TV value was 23 cm^3^ (median value). Three year rates of locoregional control were 81% and 48% for TV <23 cm^3^ and ≥23 cm^3^ (p = 0.03). At the multivariate analysis the TV remained an independent determinant of LC and OS while T-stage did not. For each 10 cm^3^ increase in TV, the relative risk of locoregional failure increases with about 30% [24]. In a Greek-German collaborative study by Kurek *et al*. the TV was negatively associated with survival with an increase in the relative risk of 6% per 10 cm^3^. Also in this study pretherapeutic TV was prognostic while TNM classification was not [39]. Plataniotis *et al*. presented prognostic impact of tumor volumetry in slightly different way reporting the significant influence of total gross tumor volume TGTV (primary gross TV (PGTV) + nodal tumor). For the whole group of patients, the median PGTV was 14.7 cm^3^, median nodal tumor was 3.7 cm^3^ and median TGTV was 25.8 cm^3^. A prognostic threshold was detected at 22.8 cm^3^. Patients with a TGTV of < 22.8 cm^3^ were more likely to achieve a completely respond and had a median survival of 45.3 months, and those with a TGTV >22.8 cm^3^ had a median survival of 12.3 months (p = 0.01) [28].

Probably the largest study reporting prognostic value of TV involved 360 patients with advanced HNC treated with CHRT. The median TV was 28.7 cm^3^. There was a significant effect of TV on LC and OS in multivariate analysis. The HR for the local recurrence and OS increased by 14% per 10 cm^3^ volume increase. T-stage remained not significantly related to treatment outcome status. The TV of 30 cm^3^ was discussed as the proposal of cut-off value for advanced tumors (T3T4) to serve as a tool for individualizing treatment [6]. The large group of 340 patients with oropharyngeal cancer was reported by Lok *et al*. The majority of patients underwent CHRT. In univariate analysis TV and T-stage significantly correlated with local failure. On multivariate analysis both TV and N-stage were independent risk factors for overall survival and distant control [27].

Early prediction of potential treatment failure after CHRT is an important goal, as salvage surgery or radiosurgery may be the only subsequent curative option and should be performed as early as possible. Bhatia *et al*. assessed the prediction of TV based on MRI examination performed at diagnosis, two weeks into and 6 weeks after CHRT on treatment outcome in 69 patients. Those with local failure had higher TV comparing to those with local remission at diagnosis (p = 0.01), 2 weeks into CHRT (p = 0.009) and 6 weeks after CHRT (p < 0.0001). Patients with local failure had smaller percentage reduction in TV 2 weeks into CHRT (p = 0.02) and 6 weeks after CHRT (p = 0.001). Authors concluded that TV based on MRI 6 weeks after completing CHRT is most predictive of local failure and, although can not be used to modify treatment, it could be important for selecting patients for more aggressive monitoring [40].

#### Prediction of regional spread

Few authors found that the regional control is well predicted by TV [5,41]. Studer *et al*. found that 2-year nodal control was 95%, 90% and 75% for the following TV ranges: 1-15 cm^3^, >15-70 cm^3^ and >70 cm^3^ respectively (p = 0.04) [5]. Certainly there is a correlation between TV and the risk of nodal metastases. It was confirmed that patients with pharyngeal tumors [42] and laryngeal tumors [13] presenting nodal metastases had significantly higher TV as compared those without nodal involvment. No such correlation was found for patients with oral or maxillary sinus cancer [42]. It was also found that for T2 epiglottic cancer the risk of nodal spread increase rapidly with TV, doubling for TV between 2.57 cm^3^ and 5.62 cm^3^, and may reach 80% for large TV (>9.70 cm^3^) [Rutkowski, unpublished data].

#### Prediction of distant metastases

Some data indicate that larger TV may increases the risk of distant spread of disease. Suwinski *et al*. re-analyzed several numerical data of TV and the risk of developing metastases that had been published in the literature (8707 cases). The results indicate that there is a threshold TV and if exceeded, the risk of developing metastases raises proportionally to the logarithm of its volume that is proportionally to the number of TV doublings. The steepest increase in the incidence of metastases per one TV doubling corresponds to TV related to about 50% incidence of metastases. In homogenous sub-groups of tumors of a particular histological type, the threshold curves for the incidence of metastases as a function of TV are steep and small increase in TV may cause large increase in the risk of metastases [43]. Studer *at al*. reported that distant spread was significantly predicted by TV, while non-significantly stratified by the T-classification or AJCC staging. Eighty eight per cent of distant metastases appeared in patients with TV >70 cm^3^[5]. In another analysis of that group of patients where the criterion of prediction was tested particularly for distant metastases, TV was the strongest predictor of it. Using similar threshold as in previous study, distant metastases appeared in 4% and 25% of patients respectively in those with tumors smaller and larger than 70 cm^3^[33]. Strongin *et al*. found that patients with locoregional failure had TVs an average of 27.1 cm^3^ larger than the TV of patients whose first site of failure was metastatic disease (58.0 cm^3^ vs. 31.0 cm^3^ p = 0.01) [7]. In the large series of 360 patients treated with CHRT the HR for distant metastases increased by 14% (95% CI, 5-24) per 10 cm^3^ TV increase [6].

### Relations between TV and other prognostic and predictive factors

Although there is an evidence that tumor hypoxia adversely affects locoregional tumor control and survival in HNC patients it is still not well established how hypoxic fraction of the tumor could be measured. Results of some studies suggest that TV may, in some way, predict effectiveness of RT both, reflecting status of tumor oxygenation and correlating with hemoglobin (Hb) concentration.

Hb concentration is well recognized prognostic factor for HNC patients treated with RT. Among other assumptions, it has been proposed that Hb is a surrogate marker for tumor hypoxia but still few data exist to test this hypothesis. Nordsmark *et al*. found both, Hb and tumor hypoxia as significant but independent prognostic factors for locoregional tumor control for HNC patients after RT, and Hb concentration was not a surrogate marker of tumor hypoxia [44]. Rutkowski *et al*. described significant negative correlation between TV and Hb concentration both, before and after RT in 160 patients with T2 laryngeal cancer [13]. Stadler *et al*. tried to reevaluate the prognostic significance of the classical oxygenation parameters like hypoxic fraction, percentage of pO_2_, median pO_2_. Additionally, he defined a new radiobiological factor – hypoxic TV (hTV) as the percentage of pO_2_-value below 5 mmHg multiplied by the TV. The rational of this parameter was to quantify approximately the amount of hypoxic tissue which should be correlated to the total number of hypoxic cells in the tumor. Pretreatment pO_2_ was assessed in 59 patients with HNC who underwent RT or CHRT. Results suggested that the total amount of hypoxic tissue determined by the hTV influenced the prognosis of patients suffering from HNC. In this study significant correlation between Hb concentration and TV was also found [45]. Continuing this idea, Dunst *et al*. defined nonhypoxic TV as a difference between the TV and hTV. It appeared that patients who were alive at analysis had significantly lower mean TV: 34 cm^3^ v 54 cm^3^ (p = 0.01) and mean hTV: 11 cm^3^ v 22 cm^3^ (p = 0.009) than those who died. In a multivariate analysis the hTV was strong and independent prognostic factor for survival (p = 0.001) and more important than TV (p = 0.02) whereas the nonhypoxic TV had no impact on prognosis (p = 0.3) [46]. Experimental data have demonstrated that the hypoxic cells can be found around or in necrosis [47]. Due to this, visible necrosis may serve as a surrogate marker for intratumor hypoxia. Significant, positive correlation between necrotic TV (nTV) and TV was reported by Kuhnt *et al*. He found that nTV based on CT scans was significantly lower for controlled patients, than for those with recurrent tumor (p = 0.003) in the group of patients treated with RT alone. Tumors with smaller amount of necrosis (<4 cm^3^) had a good prognosis regardless of the type of treatment. Patients with tumors with a larger amount of necrosis (≥4 cm^3^) had a significantly better outcome if treated with CHRT as compared to RT alone [48].

Some data indicate that TV may reflect tumor type and, in some way, general status of the patients. Rutkowski *et al*. found that T2 laryngeal tumors histologically poorly differentiated (G2,G3) presented with significantly higher median TV compared to G1 tumors (2.7 cm^3^ v 1.2 cm^3^, p = 0.001). Additionally, patients with duration of disease symptoms prior to RT longer than 6 months had significantly lower median TV comparing to others (1.6 cm^3^ v 3.8 cm^3^, p = 0.01). Significant, negative correlation between TV and weight of the patients in this group both, before and after RT was also reported [13].

Kats *et al*. investigated the association between TV, that had been diagnosed on preoperative, diagnostic CT scans, with pathologic evidence of thyroid cartilage penetration in 94 patients who undergone total laryngectomy due to cancer of the larynx. Among 49 noniradiated previously patients the mean TV of those with and without thyroid cartilage penetration was 60.1 cm^3^ and 28 cm^3^ respectively (p = 0.004). When the nonirradiated patients were divided into three TV groups (<25 cm^3^, 25-50 cm^3^, >50 cm^3^) the rates of thyroid cartilage penetration were 23%, 17% and 78% respectively (p = 0.003). No such correlation was found however for patients previously irradiated. Additionally, when patients were divided by laryngeal subsites only supraglottic tumors retained statistically significant association between volume subgroup and thyroid cartilage penetration (p = 0.04). Authors recommended incorporation of TV assessment into the diagnostic formula for predicting thyroid cartilage penetration and selecting patients who have a lower chance of benefiting from laryngeal preservation [49].

## Conclusion

It is noticeable that considering TV, a single T stage category cover tumors of various prognosis. Although it is not possible to define general prognostic cut-off value of TV for all HNC sites and stages, almost all of the clinical data sets indicate its association with RT outcome. The concept of significant influence of TV on RT results is not new, but some innovative interpretation of it may be employed in clinical practice at present. Progress in tumor visualization techniques that are used for RT planning facilitated TV evaluation. More accurate TV estimation may serve as a additional predictor between conventional RT alone, modern, highly conformal RT techniques or more aggressive, combined treatment. In any way, TV assessment should supplement clinical decision in the choice of optimal treatment strategy for HNC patients.

## Competing interests

The author declares that he has no competing interests.

## References

[B1] Johnson CR Thames HD Huang DT Schmidt-Ullrich RK The tumor volume and clonogen number relationship: tumor control predictions based upon tumor volume estimates derived from computed tomography Int J Radiat Oncol Biol Phys 1995 33 281 287 10.1016/0360-3016(95)00119-J 7673015

[B2] Brenner DJ Dose, volume, and tumor-control predictions in radiotherapy Int J Radiat Oncol Biol Phys 1993 26 171 179 10.1016/0360-3016(93)90189-3 8482624

[B3] AJCC Cancer Staging Manual 7 New York Dordrecht Heidelberg London: Springer 201

[B4] Johnson CR Khandelwal SR Schmidt-Ullrich RK Ravalese J 3rd Wazer DE The influence of quantitative tumor volume measurements on local control in advanced head and neck cancer using concomitant boost accelerated superfractionated irradiation Int J Radiat Oncol Biol Phys 1995 32 635 641 10.1016/0360-3016(95)00031-S 7790249

[B5] Studer G Lütolf UM El-Bassiouni M Rousson V Glanzmann C Volumetric staging (VS) is superior to TNM and AJCC staging in predicting outcome of head and neck cancer treated with IMRT Acta Oncol 2007 46 386 394 10.1080/02841860600815407 17450476

[B6] Knegjens JL Hauptmann M Pameijer FA Balm AJ Hoebers FJ de Bois JA Kaanders JH van Herpen CM Verhoef CG Wijers OB Wiggenraad RG Buter J Rasch CR Tumor volume as prognostic factor in chemoradiation for advanced head and neck cancer Head Neck 2011 33 375 382 20629076 10.1002/hed.21459

[B7] Strongin A Yovino S Taylor R Wolf J Cullen K Zimrin A Strome S Regine W Suntharalingam M Primary tumor volume is an important predictor of clinical outcomes among patients with locally advanced squamous cell cancer of the head and neck treated with definitive chemoradiotherapy Int J Radiat Oncol Biol Phys 2012 82 1823 1830 10.1016/j.ijrobp.2010.10.053 21549516

[B8] Sorensen AG Patel S Harmath C Bridges S Synnott J Sievers A Yoon YH Lee EJ Yang MC Lewis RF Harris GJ Lev M Schaefer PW Buchbinder BR Barest G Yamada K Ponzo J Kwon HY Gemmete J Farkas J Tievsky AL Ziegler RB Salhus MR Weisskoff R Comparison of diameter and perimeter methods for tumor volume calculation J Clin Oncol 2001 19 551 557 11208850 10.1200/JCO.2001.19.2.551

[B9] Kraas JR Underhill TE D'Agostino RB Jr Williams DW 3rd Cox JA Greven KM Quantitative analysis from CT is prognostic for local control of supraglottic carcinoma Head Neck 2001 23 1031 1036 10.1002/hed.10030 11774387

[B10] Pameijer FA Balm AJ Hilgers FJ Muller SH Variability of tumor volumes in T3-staged head and neck tumors Head Neck 1997 19 6 13 10.1002/(SICI)1097-0347(199701)19:1<6::AID-HED2>3.0.CO;2-A 9030938

[B11] Studer G Glanzmann C Volumetric stratification of cT4 stage head and neck cancer Strahlenther Onkol 2013 189 867 873 10.1007/s00066-013-0413-3 24002381 PMC3825283

[B12] Nathu RM Mancuso AA Zhu TC Mendenhall WM The impact of primary tumor volume on local control for oropharyngeal squamous cell carcinoma treated with radiotherapy Head Neck 2000 22 1 5 10.1002/(SICI)1097-0347(200001)22:1<1::AID-HED1>3.0.CO;2-6 10585598

[B13] Rutkowski T Wygoda A Składowski K Hejduk B Rutkowski R Kołosza Z Maciejewski B Prognostic role of tumor volume for radiotherapy outcome in patient with T2 laryngeal cancer Strahlenther Onkol 2013 189 861 866 10.1007/s00066-013-0411-5 23982169

[B14] Pameijer FA Mancuso AA Mendenhall WM Parsons JT Mukherji SK Hermans R Kubilis PS Evaluation of pretreatment computed tomography as a predictor of local control in T1/T2 pyriform sinus carcinoma treated with definitive radiotherapy Head Neck 1998 20 159 168 10.1002/(SICI)1097-0347(199803)20:2<159::AID-HED10>3.0.CO;2-H 9484948

[B15] Sze WM Lee AW Yau TK Yeung RM Lau KY Leung SK Hung AW Lee MC Chappell R Chan K Primary tumor volume of nasopharyngeal carcinoma: prognostic significance for local control Int J Radiat Oncol Biol Phys 2004 59 21 27 10.1016/j.ijrobp.2003.10.027 15093895

[B16] Pameijer FA Mancuso AA Mendenhall WM Parsons JT Kubilis PS Can pretreatment computed tomography predict local control in T3 squamous cell carcinoma of the glottic larynx treated with definitive radiotherapy? Int J Radiat Oncol Biol Phys 1997 37 1011 1021 10.1016/S0360-3016(96)00626-8 9169807

[B17] Hermans R Feron M Bellon E Dupont P Van den Bogaert W Baert AL Laryngeal tumor volume measurements determined with CT: a study on intra- and interobserver variability Int J Radiat Oncol Biol Phys 1998 40 553 557 10.1016/S0360-3016(97)00853-5 9486604

[B18] Mancuso AA Mukherji SK Schmalfuss I Mendenhall W Parsons J Pameijer F Hermans R Kubilis P Preradiotherapy computed tomography as a predictor of local control in supraglottic carcinoma J Clin Oncol 1999 17 631 637 10080608 10.1200/JCO.1999.17.2.631

[B19] Maroldi R Battaglia G Farina D Maculotti P Chiesa A Tumours of the oropharynx and oral cavity: perineural spread and bone invasion JBR-BTR 1999 82 294 300 10670171

[B20] Daisne JF Duprez T Weynand B Lonneux M Hamoir M Reychler H Grégoire V Tumor volume in pharyngolaryngeal squamous cell carcinoma: comparison at CT, MR imaging, and FDG PET and validation with surgical specimen Radiology 2004 233 93 100 10.1148/radiol.2331030660 15317953

[B21] Vanuytsel LJ Vansteenkiste JF Stroobants SG De Leyn PR De Wever W Verbeken EK Gatti GG Huyskens DP Kutcher GJ The impact of (18)F-fluoro-2-deoxy-D-glucose positron emission tomography (FDG-PET) lymph node staging on the radiation treatment volumes in patients with non-small cell lung cancer Radiother Oncol 2000 55 317 324 10.1016/S0167-8140(00)00138-9 10869746

[B22] Allal AS Slosman DO Kebdani T Allaoua M Lehmann W Dulguerov P Prediction of outcome in head-and-neck cancer patients using the standardized uptake value of 2-[18 F]fluoro-2-deoxy-D-glucose Int J Radiat Oncol Biol Phys 2004 59 1295 1300 10.1016/j.ijrobp.2003.12.039 15275712

[B23] Seol YM Kwon BR Song MK Choi YJ Shin HJ Chung JS Cho GJ Lee JC Lee BJ Wang SG Kim HJ Kim WT Kim SJ Yun EY Measurement of tumor volume by PET to evaluate prognosis in patients with head and neck cancer treated by chemo-radiation therapy Acta Oncol 2010 49 201 208 10.3109/02841860903440270 20100156

[B24] Hoebers FJ Pameijer FA de Bois J Heemsbergen W Balm AJ Schornagel JH Rasch CR Prognostic value of primary tumor volume after concurrent chemoradiation with daily low-dose cisplatin for advanced-stage head and neck carcinoma Head Neck 2008 30 1216 1223 10.1002/hed.20865 18642295

[B25] Bellon E Op de beeck K Feron M Staelens L Rijnders A Van den Bogaert W Hermans R The relation of CT-determined tumor parameters and local and regional outcome of tonsillar cancer after definitive radiation treatment Int J Radiat Oncol Biol Phys 2001 50 37 45 10.1016/S0360-3016(00)01559-5 11316544

[B26] Rudat V Dietz A Schramm O Conradt C Maier H Flentje M Wannenmacher M Prognostic impact of total tumor volume and hemoglobin concentration on the outcome of patients with advanced head and neck cancer after concomitant boost radiochemotherapy Radiother Oncol 1999 53 119 125 10.1016/S0167-8140(99)00119-X 10665788

[B27] Lok BH Setton J Caria N Romanyshyn J Wolden SL Zelefsky MJ Park J Rowan N Sherman EJ Fury MG Ho A Pfister DG Wong RJ Shah JP Kraus DH Zhang Z Schupak KD Gelblum DY Rao SD Lee NY Intensity-modulated radiation therapy in oropharyngeal carcinoma: effect of tumor volume on clinical outcomes Int J Radiat Oncol Biol Phys 2012 82 1851 1857 10.1016/j.ijrobp.2011.03.029 21640497 PMC4978948

[B28] Plataniotis GA Theofanopoulou ME Kalogera-Fountzila A Haritanti A Ciuleanou E Ghilezan N Zamboglou N Dimitriadis A Sofroniadis I Fountzilas G Prognostic impact of tumor volumetry in patients with locally advanced head-and-neck carcinoma (non-nasopharyngeal) treated by radiotherapy alone or combined radiochemotherapy in a randomized trial Int J Radiat Oncol Biol Phys 2004 59 1018 1026 10.1016/j.ijrobp.2004.01.021 15234035

[B29] Chen SW Yang SN Liang JA Lin FJ Tsai MH Prognostic impact of tumor volume in patients with stage III-IVA hypopharyngeal cancer without bulky lymph nodes treated with definitive concurrent chemoradiotherapy Head Neck 2009 31 709 716 10.1002/hed.21011 19260114

[B30] Lo SM Venkatesan V Matthews TW Rogers J Tumour volume: implications in T2/T3 glottic/supraglottic squamous cell carcinoma J Otolaryngol 1998 27 247 251 9800621

[B31] Hermans R Van den Bogaert W Rijnders A Doornaert P Baert AL Predicting the local outcome of glottic squamous cell carcinoma after definitive radiation therapy: value of computed tomography-determined tumor parameters Radiother Oncol 1999 50 39 46 10.1016/S0167-8140(98)00114-5 10225556

[B32] Hermans R Van den Bogaert W Rijnders A Baert AL Value of computed tomography as outcome predictor of supraglottic squamous cell carcinoma treated by definitive radiation therapy Int J Radiat Oncol Biol Phys 1999 44 755 765 10.1016/S0360-3016(99)00039-5 10386632

[B33] Studer G Seifert B Glanzmann C Prediction of distant metastasis in head neck cancer patients: implications for induction chemotherapy and pre-treatment staging? Strahlenther Onkol 2008 184 580 585 10.1007/s00066-008-1951-y 19016016

[B34] Chua DT Sham JS Kwong DL Tai KS Wu PM Lo M Yung A Choy D Leong L Volumetric analysis of tumor extent in nasopharyngeal carcinoma and correlation with treatment outcome Int J Radiat Oncol Biol Phys 19971 39 711 719 9336154 10.1016/s0360-3016(97)00374-x

[B35] Hamilton S Venkatesan V Matthews TW Lewis C Assis L Computed tomographic volumetric analysis as a predictor of local control in laryngeal cancers treated with conventional radiotherapy J Otolaryngol 2004 33 289 294 10.2310/7070.2004.03088 15931812

[B36] Mukherji SK Mancuso AA Mendenhall W Kotzur IM Kubilis P Can pretreatment CT predict local control of T2 glottic carcinomas treated with radiation therapy alone? AJNR Am J Neuroradiol 1995 16 655 662 7611018 PMC8332275

[B37] Rutkowski T Accepted Article

[B38] Studer G Glanzmann C Volumetric staging in oropharyngeal cancer patients treated with definitive IMRT Oral Oncol 2013 49 269 276 10.1016/j.oraloncology.2012.09.014 23089460

[B39] Kurek R Kalogera-Fountzila A Muskalla K Dafni U Schnabel T Kober B Röddiger S Martin T Fountzilas G Zamboglou N Usefulness of tumor volumetry as a prognostic factor of survival in head and neck cancer Strahlenther Onkol 2003 179 292 297 10.1007/s00066-003-1017-0 12740655

[B40] Bhatia KS King AD Yu KH Vlantis AC Tse GM Mo FK Ahuja AT Does primary tumour volumetry performed early in the course of definitive concomitant chemoradiotherapy for head and neck squamous cell carcinoma improve prediction of primary site outcome? Br J Radiol 2010 83 964 970 10.1259/bjr/27631720 20965907 PMC3473721

[B41] Grabenbauer GG Steininger H Meyer M Fietkau R Brunner T Heinkelmann P Hornung J Iro H Spitzer W Kirchner T Sauer R Distel L Nodal CT density and total tumor volume as prognostic factors after radiation therapy of stage III/IV head and neck cancer Radiother Oncol 1998 47 175 183 10.1016/S0167-8140(98)00016-4 9683366

[B42] Kimura Y Sumi M Ichikawa Y Kawai Y Nakamura T Volumetric MR imaging of oral, maxillary sinus, oropharyngeal, and hypopharyngeal cancers: correlation between tumor volume and lymph node metastasis AJNR Am J Neuroradiol 2005 26 2384 2389 16219850 PMC7976164

[B43] Suwiński R Withers HR Ocena zależności między objętością guza pierwotnego a ryzykiem wystąpienia przerzutów i jej implikacje kliniczne Nowotwory 1999 49 513 521

[B44] Nordsmark M Overgaard J Tumor hypoxia is independent of hemoglobin and prognostic for loco-regional tumor control after primary radiotherapy in advanced head and neck cancer Acta Oncol 2004 43 396 403 10.1080/02841860410026189 15303502

[B45] Stadler P Becker A Feldmann HJ Hänsgen G Dunst J Würschmidt F Molls M Influence of the hypoxic subvolume on the survival of patients with head and neck cancer Int J Radiat Oncol Biol Phys 1999 44 749 754 10.1016/S0360-3016(99)00115-7 10386631

[B46] Dunst J Stadler P Becker A Lautenschläger C Pelz T Hänsgen G Molls M Kuhnt T Tumor volume and tumor hypoxia in head and neck cancers. The amount of the hypoxic volume is important Strahlenther Onkol 2003 521 526 14509950 10.1007/s00066-003-1066-4

[B47] Ljungkvist AS Bussink J Rijken PF Kaanders JH van der Kogel AJ Denekamp J Vascular architecture, hypoxia, and proliferation in first-generation xenografts of human head-and-neck squamous cell carcinomas Int J Radiat Oncol Biol Phys 2002 54 215 228 12182995 10.1016/s0360-3016(02)02938-3

[B48] Kuhnt T Mueller AC Pelz T Haensgen G Bloching M Koesling S Schubert J Dunst J Impact of tumor control and presence of visible necrosis in head and neck cancer patients treated with radiotherapy or radiochemotherapy J Cancer Res Clin Oncol 2005 131 758 764 10.1007/s00432-005-0018-z 16088405 PMC12161190

[B49] Kats SS Muller S Aiken A Hudgins PA Wadsworth JT Shin DM Khuri F Beitler JJ Laryngeal tumor volume as a predictor for thyroid cartilage penetration Head Neck 2013 35 426 430 10.1002/hed.22995 22488941

